# Designing an Interactive Field Epidemiology Case Study Training for Public Health Practitioners

**DOI:** 10.3389/fpubh.2018.00275

**Published:** 2018-09-26

**Authors:** Amy L. Nelson, Lauren Bradley, Pia D. M. MacDonald

**Affiliations:** RTI International, Research Triangle Park, NC, United States

**Keywords:** epidemiology, public health, workforce development, training, case study

## Abstract

Globally, public health practitioners are called upon to respond quickly and capably to mitigate a variety of immediate and incipient threats to the health of their communities, which often requires additional training in new or updated methodologies or epidemiologic phenomena. Competing public health priorities and limited training resources can present challenges in developed and developing countries alike. Training provided to front-line public health workers by ministries of health, donors and/or partner organizations should be delivered in a way that is effective, adaptable to local conditions and culture, and should be an experience perceived as a job benefit. In this review, we share methods for interactive case-study training methodologies, including the use of problem-based scenarios, role-play activities, and other small-group focused efforts that encourage the learner to discuss and synthesize the concepts taught. We have fine-tuned these methods through years of carrying out training of all levels of public health practitioners in dozens of countries worldwide.

## Background and rationale

In a rapidly changing field marked by frequent turnover, the need to provide continuing education for public health practitioners is constant. Often there is no requirement for prior credentialing in public health for local-level jobs^1^,^2^. Alternatively, training and professional development may be clinically-oriented as opposed to the more relevant population based public health focus (1). In many parts of the world public health training programs targeting the health professions are minimal (2). For these reasons, in the US and elsewhere, public health professions tend to be high-responsibility, low-pay jobs, marked by high turnover rates (3).

In many low- and middle-income countries, keeping educated health professionals in-country is challenging (4). Public health professionals are discouraged by lack of proper compensation and insufficient opportunities for continuing professional development and education (5). Results from a 2006 study conducted for the Ugandan Ministry of Health investigating health workforce morale, satisfaction, motivation, and intent to remain in Uganda showed that health workers were dissatisfied with their jobs. About one in four noted a desire to leave the country to improve their outlook (6). Further complicating matters, those who have learned occupational skills through on-the-job experience are vulnerable to being laid off with changes of government administration (7).

Our experience carrying out training activities over the last two decades has provided the opportunity to train public health practitioners in-person, online, and via self-directed learning. We have designed and conducted trainings for front-line local responders as well as district and national-level officials both in the US and abroad. For example, in the US, we have trained county and state-level public health workers in surveillance and outbreak investigation through in-person training courses, Web conferencing platforms, and via both synchronous and asynchronous distance-based methods. We have trained national-level officials and laboratorians through specialized in-person trainings as well as via large conference-based and train-the-trainer style settings. Examples include organizing and developing content for a multi-agency tabletop exercise focused on bioterrorism response post-9/11, development of a curriculum focused on rapid response to avian and pandemic influenza, strengthening workforce capacity in sentinel site surveillance for respiratory illness, and, with the US Centers for Disease Control and Prevention (CDC), a full Master's-degree level curricula the Central American Field Epidemiology Training Program (FETP) Master's degree program, as well as front-line public health practitioner trainings at Intermediate and Basic-levels within Central America and topic-specific FETP modules elsewhere. These efforts were supported by CDC, the National Council of State and Territorial Epidemiologists (CSTE), the World Health Organization (WHO), the Pan-American Health Organization (PAHO), and others.

Whether delivered to a non-epidemiologist who finds themselves responsible for outbreak investigation, or to a national epidemiology official who will subsequently oversee training of their staff, the case study or interactive exercise typically serves as a pivotal feature of training for public health professionals, moving them toward actively engaging in learning, knowledge sharing and in the application of previously learned information (8). We describe key elements in the development and use of case studies as a learning tool for public health practitioners, based on our years of experience in developing, implementing, and evaluating public health trainings. The learning objectives of this article are the following:

- Identify critical teaching points around which to structure a case study training.
- Consider relevant sources of information to provide information and “plot” to a case study.
- Choose a case study format appropriate for the trainees and content.
- Incorporate design elements that encourage interaction and thoughtful discussion among learners into a case study.

## Pedagogical framework

Case-study based learning approaches have proven particularly effective in the medical education context. Comparison studies conducted in the medical sciences have found interactive, case-study focused learning a more effective teaching tool than didactic lectures (9, 10). Although the literature focuses on medical education, the success of case studies in public health education is also reflected in the growing number of public health programs with problem-based curricula (11, 12). Our goal has been to create effective training materials for public health professionals that present relevant, interactive, and interesting content that teach job skills (not merely general principles) in a way that can be quickly understood and applied while providing an opportunity for peers of different experience levels to learn from one another.

Curriculum development is a multi-layered process involving instructional designers, subject matter experts, and editorial and art personnel (13). Training format is highly dependent upon the needs of the involved partner(s). Information-driven training presented in the didactic style only can be less effective for information retention and learner motivation. Learning methods allowing the participant to contribute meaningfully from their own experience, develop skills in a supervised setting, and practice skills immediately pursuant to learning them, will result in more retention and ability to apply the skills taught. Learners who are given ample opportunities to analyze past experience through reflection, evaluation and reconstruction fulfill a key element of experience-based learning and are able to draw deeper meaning from prior events in their personal and professional lives (14). As such, interactive and problem-based approaches to building applied public health skills are critical features of many public health trainings. Interactive and problem-based approaches engender openness toward new experiences, a critical element in facilitating lifelong learning. At the social or group level, these approaches help emphasize critical social action and the importance of adopting a stance shaped by moral and socio-political responsibility (14).

### Setting

Dissemination of information or didactic methods may be used as a means of communicating concepts, methods, or the use of new technologies, but should be accompanied by interactive teaching methods. Interactive teaching can occur in a variety of settings, which may be dependent on program, resources, and learner characteristics. The training audience is professional public health practitioners, who may or may not be formally educated in public health.

The size of the training has varied vastly, from fewer than 12 learners to over 100. With larger groups, we often have a lead instructor who is experienced both in the subject area and in carrying out trainings who is accompanied by “facilitators” or small-group leaders who can provide guidance during case-study work in break-out sessions.

### Format

Although we have designed trainings in many different formats, the most successful setting for a case study in our experience has been one where learners come together in-person for a scheduled period of instruction and worked activities, ranging in time from half-day trainings to 2-week courses.

### Evaluation

The success of a particular training methodology is difficult to assess. For the several dozen trainings we have carried out, we include a participant evaluation that captures information such as whether the participant found the training useful, whether they feel able to use the information learned in their job, how satisfied they were with the training, and open comments. Informal interaction with trainees and feedback from these surveys over the years has shaped the case study format presented here. The success of a public health training should be measurable by participants' ability to perform specific job functions, and more broadly, improvements in components of the public health system such as timeliness and completeness of communicable disease reporting and surveillance.

## Blueprints for an interactive curriculum

There are several considerations when planning and developing curriculum materials. First, the needs of the intended target audience (i.e., the learners) should form the core of any development process. The curriculum should be designed to both meet training and skill-building needs, and to approach the target audience in a comfortable and accessible way; it should meet them at their current level; the materials should address the fact that—trained or not—professionals who have been working in any field have experience to contribute that adds to the depth of a training. Second, training materials should be developed in a way that ensures consistency in the delivery. Thus, lectures should include a speaker script and built-in questions for the audience with suggestions to both engage learners and continually check knowledge acquisition. Having the script provides particular flexibility for train-the-trainer programs or other circumstances where the person with the most content and training expertise may not be the one to personally deliver the content to all trainees. Case studies should be developed in a standard format that includes an instructor guide complete with suggested answers, worked calculations, and suggested instructional methods for emphasizing critical teaching points. These methods ensure that critical content is taught regardless of the depth of experience of the teacher or facilitator. Finally, training materials should engage the learner in discussion and healthy debate, contribute to their own learning, and provide peer-to-peer learning opportunities. We discuss several types of interactive exercises which are often collectively referred to as “case studies,” but specifically may feature different elements (Table 1).

**Table 1:** Terminology, definition, and examples of interactive training activities often referred to as “case studies.”

**Terminology**	**Definition**	**Example(s)**
Exercise	A short Q&A (~5 min) between instructor and the class that is embedded in didactic lectures or presentations	In a biostatics lecture about probability distributions, the instructor asks every student to take out a coin, flip it 10 times and note how many heads and tails they obtained, and then report the number of heads. The instructor graphs these results on a histogram at the front of the class to demonstrate binomial distributions
Activity	A project accomplished in small groups where students answer questions and develop and apply concepts that were taught in a didactic lecture. These are often scenario-based but may take other formats	• Analyze a dataset from an epidemiologic investigation • Critique an abstract or manuscript • Develop public health surveillance priorities • Create a data collection instrument to be used for a rapid needs assessment
Tabletop	A scenario-based exercise that facilitates inter-agency collaboration, often in the context of planning for state or national emergencies such as disasters, pandemics, or bioterrorism. May or may not be accompanied by a didactic component	Professionals from public health, agriculture, wildlife, the judiciary, customs, transportation, and/or law enforcement gather at the table to play their respective roles in a scenario where illegal importation of chickens leads to an outbreak of a new, highly pathogenic strain of influenza A on a domestic poultry farm
Case study	Any form of scenario-based work in which information is gradually released to the learner as they progress through the scenario. Learners are directed to apply core training concepts as they move along. May be designed for individuals, small groups, or breakout groups from a larger classroom setting	Learners are given background information and must assess whether to investigate an outbreak. Subsequently they progress through the steps of an outbreak investigation as they are given “updates” on how many cases are occurring and clues they find through their investigation

We propose the following general objectives of a group case study, regardless of format or topic area:

- To meet defined learning objectives or competencies.
- To learn in an environment that respects the skills and abilities of the learners, and allows each learner to use and apply their unique skills.
- To have learners interact with each other, thus enhancing their own learning through listening to the ideas and experience of fellow learners.
- To exhibit genuine interest in and be motivated by the content they are learning.

We have found that there are four critical conceptual elements in developing and executing an interactive training:

1. Reference key points or concepts to be learned and applied.
2. Guide learners to work through a problem.
3. Keep learners engaged through active questioning.
4. Draw on learners' experience.

Each of these is described in detail below, with a measles outbreak in a small African village used as a guide for demonstrating their implementation through a case study exercise.

### Reference key points or concepts to be learned and applied

The main intent of a case study is to teach predefined content. Often training delivery of a case study is preceded by didactic content, or other information-driven training such as web-based tutorials, workbooks, or reading material. Whether or not information-driven training is included, the key concepts to be learned and applied should be viewed as the framework upon which the rest of the case study will be built. For example, a lecture or reading may put forth a set of 9 steps to investigate an outbreak. The case study format will then be dictated by achieving each of these 9 steps. It is of benefit to clearly and obviously delineate any key steps or phases as one works through the case study. The beginning learner gains more benefit from understanding a firm case study structure than from trying to figure out which step or concept should be applied in which situations, even though events may not occur so neatly on the job. When teaching a set of concepts, it is better to teach them clearly and simply than to allow the learner to become frustrated, struggle, and possibly fumble. More advanced learners can be given the opportunity to struggle with decision-making and unexpected sequences of events, after they have mastered a clear set of tools from which to draw. A few examples of clearly structured concepts that lend themselves to case studies are given in Table 2, and a scenario using these types of structures within a case study is given in Table 3.

**Table 2:** Examples of concepts that can be given a clear sequence or structure for teaching purposes.

**Sequence-based Teaching Concepts**
• Steps to conduct an outbreak investigation • Designing a study to investigate a health problem • Process for evaluating a surveillance system • Methods for communicating with the public and the media • Steps of writing an analysis plan • Methods to assess data for confounding and effect measure modification • Strategy for writing and delivering an oral presentation at a scientific conference

**Table 3:** Scenario-based examples of a measles outbreak in a small village case study.

**Topic**	**Content**
(A) Give clear learning objectives to provide a blueprint for the case study	After completing this case study, the participant should be able to: • List the early steps of an outbreak investigation • Describe several clinical diseases that have a similar presentation and how to differentiate them • Create a line list for an outbreak investigation • Calculate percentages for different symptoms of disease • Identify factors that can influence a community's willingness to participate in public health programs
(B) Provide information for the scenario	**Background** It is Thursday, September 15th. You are an epidemiologist working in the Ministry of Health (MoH). As you are nearing the end of your workday, you receive a phone call. The caller is part of a vaccination team that has just arrived in “Gwema” Village as part of an effort to vaccinate the children of the prefecture. This prefecture has been experiencing rising numbers of preventable infectious diseases, and the vaccination campaign is considered a vital step in slowing this trend. Apparently, however, residents heard that in another prefecture, vaccination campaigns were followed by cases of Ebola. Thus the vaccination team is being met with a certain degree of suspicion. On top of this, the small Gwema primary school reportedly has 6 children out of 81 total children who have become ill with a sudden fever. A MoH staff member, Dr. Miriama Daillo, is currently close to Town A and is dispatched to investigate the cases of fever. You are responsible for maintaining communications with Dr. Daillo and offering her advice and guidance by phone.
(C) Active questioning with answers for the instructor	**Question:** What are the first steps you would advise Dr. Daillo to take to determine whether an outbreak is really occurring? ***Instructor Answer:*** 1. Establish the existence of an outbreak, and 2. Verify the diagnosis. A good start is to interview cases: Determine whether all cases have the same illness; Determine whether the illness is unusual for the time, place, and population group.
(D) Draw on personal and professional experience	**Question:** In your own role (health department/job/experience), have you ever had to initiate a vaccination campaign for measles or another vaccine-preventable illness? If so, who did you work with? What steps did you take? What challenges did you face? Was it successful? Why or why not? What would you do differently in a similar situation next time? **Question:** Given the information that you have received about this measles outbreak in Gwema Village, do you think is it too late to implement control measures such as a vaccination campaign?

Regardless of topic, a simple introduction for the participant and the instructor as to the point and process of the case study is required. Begin by providing learning objectives (as in Table 3A) and a short introduction to the case study, including if, and how, learners should interact with others, the resources they should use for case study completion, the product that is expected, and the estimated time required to complete the tasks given.

### Guide learners to work through a problem

A hallmark feature of a case study is working through a problem that parallels or is based on a real-life situation. An appealing format is to present the learner with introductory or background information and update information as the learner works through the problems of the case study.

Basing information and scenarios on real events is the best way to prevent the scenario from feeling contrived. Possible sources of information for scenarios include the experience, internal reports, or data of the curriculum developer or colleagues; published literature; local, national, or international bulletins; health situation updates provided by State health departments, CDC or WHO; and less formal sources such as news reports and ProMed, the Program for Monitoring Emerging Diseases. In some cases, the details of an actual outbreak, surveillance system, or other event can be used directly. Typically, an actual scenario can be used, but the details can be embellished, drawn out, or supplemented by different events in order to meet the learning purposes of the case study. For example, in a scenario where the learner is in the role of someone getting ready to plan a case-control study, the setting from a ProMed report or international bulletin might be ideal, but the details given from a case-control study published in the literature provide the topical details needed to expand upon the case study scenario. Unless teaching about a specific real-event is a learning objective, a case study developer should be free to modify details and provide creative license to developing characters for the case study events. The example case study used in Table 3 of this article was inspired by a health news article about difficulties facing vaccination campaigns in Guinea^3^, and was structured on WHO recommendations for measles surveillance and case definitions (15). Resource material used to develop the case study should be appropriately cited.

Once the learning objectives, introduction, and plot for the scenario are outlined, the learner or the group is positioned within the scenario through assignment of specific roles or responsibilities, for example:

- “You are the health director for Mlima Province, and you receive a phone call from the local hospital epidemiologist who is concerned about….”
- “You are a surveillance officer with the Forêt Health district, and you have just been placed in charge of developing a new surveillance system for…”

The plot of the case study can then be developed as questions are posed, the learner makes plans, decisions, calculations, and more. An example presentation of the scenario based on the previously established measles scenario is given in Table 3B.

### Keep learners engaged through active questioning

A key to a successful case study is placing learners in an active role, rather than asking them to reproduce information-driven training concepts in the context of the scenario. Although a case study is designed to be a classroom tool, in field epidemiology the case study should strive to put the learner in the field. For data-centered trainings, this can be accomplished by instructing the learner to carry out data processing and analysis. For example, a learner may be provided with a line listing and asked to construct an epidemic curve, or they may be provided a two-by-two table and asked to calculate relative risk. The effort to place the learner in an active role is particularly important for more routine types of questions that aim to get the learner to repeat concepts (as with an information-driven training). Some examples of inactive questions and how they can be modified to be active are shown in Table 4.

**Table 4:** Comparison of scenarios with inactive vs. active questioning methods.

**Scenario**	**Inactive**	**Active**
Public health communications	How should complicated risk information be communicated to the media?	When responding to the media, how will you ensure that accurate public health information is communicated in a way that describes the risk without inciting fear or panic?
Personal safety	What personal protective equipment should be worn when interviewing a subject with suspected or confirmed influenza A (H5N1)?	What personal protective equipment will you wear when you interview the subject with suspected influenza A (H5N1)?
Public health surveillance	Should non-traditional sources of surveillance data be included in a new influenza sentinel surveillance system? Why or why not?	What sources of non-traditional surveillance data will you incorporate into the new influenza sentinel surveillance system? Justify your choices
Outbreak investigation	Give an example of close-ended questionnaire questions that gather information on food intake history in an outbreak	Write close-ended questions for the food history section of the questionnaire that you will use to investigate this outbreak

Questions should cause the learner to use the knowledge and information they have gleaned both within and outside of the training to “act” or make a decision but should not affect the ultimate plot of the case study. See an example from the measles case study in Table 3C.

### Draw on learners' experience

Adult learners are motivated by experiences that affect them personally. Facilitating contributory participation increases their personal investment (16). For a group case study, there are multiple advantages in using the case study to draw out learners' experience and personal perspective. First, when learners contribute their own experience to the case study, they gain confidence and enjoyment throughout the activity. Second, a case study that assumes learners have no experience or contribution may feel pedantic or even condescending. Third, encouraging the learner to share their ideas, opinions, and experience creates a synergistic learning opportunity that the questions on the page alone cannot accomplish. This can be done by asking discussion or reflection questions during the progress of the exercise and at its conclusion. Questions that aim to solicit information from the learner regarding past experiences he or she has in making decisions or navigating situations like those encountered in the case study can be effective in enhancing the learning experience. These types of questions can also encourage beneficial self-reflection and allow for recognition of how skills have been or could be applied in reality. Alternatively, questions that treat issues or controversies with no perfect answer fit into the context of the case study while drawing on learners' opinions, often leading to rich dialogue and experience sharing. Table 3D provides a scenario-based example of how to draw upon learner experiences relevant to the measles case study.

## Interactive training design options

The specific design used will depend on training content, setting, and the number of learners. Below, we discuss several designs in the context of smaller groups (e.g., 9 or fewer individuals) as well as larger groups (e.g., 10–30 individuals). In many cases, larger groups can be accommodated with small-group case study designs. Using “breakout” groups, where a classroom of a couple dozen or more breaks into smaller groups for case study work, is the easiest method to conduct participatory case-study based training. In many cases, small modifications can be made that also take advantage of the larger class size.

### Problem-based scenarios

Problem-based scenarios are the mainstay of case study trainings. They guide learners through carrying out activities and can be flexible in length, depending on the depth of information covered and activities or computations required.

#### Small groups

In a problem-based scenario, a situation or background information is presented, and the individual or small group must work through a series of questions that address learning objectives in the context presented. Questions ask learners to provide information, conduct a calculation, or come to a decision and move on to the next question. Additional information that adds to the scenario may be provided once or several times throughout the case study.

#### Large groups

A couple variations on the problem-based case study scenario make them more interesting in the large group setting. One option is to create slightly different scenarios for breakout groups. For example, in a case study where groups are designing surveillance systems according to set principles taught in class, each small group can focus on a different set of diseases or conditions for their surveillance system. Alternatively, small groups can be provided with updates unique to their group. While this requires additional work in developing case study answer guides, small groups can present their results to each other at the end of the session and can learn more about public health challenges and considerations than just the work they themselves have performed.

### Role play

Role plays are excellent tools for practicing scenarios in which the learner must think of what to say or do, and work well for interview situations and meeting scenarios. They are best used among learners who have already spent some time together, so that fewer inhibitions exist.

#### Small groups

While many case studies ask the learner to assume they have a specific role or identity in order to answer case study questions, a role-play asks a group of learners to go a step further by carrying out interactions with other group members from the perspective of the role they are playing. To prevent learners from feeling uncomfortable in carrying out their role play, each “role” should contain specific guidance with information about their character, including general terms about what they should say and do in the role play.

Acting out an interview can provide inexperienced learners an opportunity to practice and offers experienced learners an opportunity to add improvisational content from their own experience. Scenarios may have learners conduct interviews with food workers, business proprietors, case-patients, research subjects, hospital personnel, and more. Additionally, they may be the interviewee with members of the media or government officials. Both the person conducting the interview and the person being interviewed are given guidance that can include questionnaires or question topic domains for the interviewer, a personality profile of their roles, background information for the person answering questions, top priority concerns for stakeholders, and any other details relevant to the situation. Additionally, role players can be given guidance to cause challenges for each other. For example, an interviewee's information sheet can tell them that they do not like to discuss “personal” problems, and they should do their best to avoid directly answering questions that ask for sensitive information.

Town hall meetings, stakeholder meetings, or media events can be simulated when roles relevant to the situation are assigned to different group members. For example, group members could be in a scenario dealing with student health, and members can be assigned the roles of school officials, parents, health department personnel, or students. Information sheets for each role can encourage the role player to be at odds with others to encourage discussion, bring up concepts taught in the class, and rationalize their actions relevant to the scenario. The value derived from role playing is in practicing personal interactions and the process of considering various viewpoints.

#### Large groups

Role plays can be carried out within the context of the large group by dividing the audience into breakout groups, each representing a group of people, such as health spokespeople, media, or community members. The entire group would have the same pre-defined list of character traits or concerns, but individuals within the group voicing their own perspective provide lively interaction. Another option is to have each small group carry out the same role play, with one person per role, and then to follow the role play with a large group discussion. The instructor can ask questions such as, “For those of you who conducted the interview, what was the most challenging aspect of obtaining the information you needed?” Debriefing encourages learners to process what they have learned and allows them to share their own experiences in similar circumstances.

### Create a common product

This exercise is particularly useful when the skills to complete a large task are being taught, such as skills for writing reports or designing surveillance systems.

#### Small groups

Although it is not feasible, in a classroom setting, to have learners complete a large task at their desk, as a group they can efficiently combine forces to cover key concepts from the teaching and produce a common paper, outline, graph, or presentation that addresses key points. Simple example assignments to a small group include:

- Given the scenario, create a flow diagram of a surveillance system that collects population-based pneumonia and influenza data.
- Write the outline of a bulletin article that summarizes the outbreak investigation methods and results that we have worked through today.
- With each group member taking responsibility for one section of a protocol, write an outline for key content to be included in each section. Then share your results with your group members and solicit their feedback.

#### Large groups

Breakout groups creating a common product can provide an added dimension and present their product to the entire class. In these cases, it is beneficial to ask the class for constructive criticism on each other's work. Audience members can rate the presenting group against how well they addressed class concepts or met defined criteria. We have used Olympic-style score cards for this task, which lessens the risk of “speaking out” from the viewpoint of the learner offering feedback, but also gives the instructor impetus to ask specific individuals for their rationale in providing that score.

### “Go out” assignments

Although the ability to use this exercise is highly dependent upon the training setting, “going out” is an interactive way to solidify concepts in questionnaire design, questionnaire execution, and data collection.

#### Small or large groups

If a training is being held in a location where learners have easy and safe access to a public lunch room, university students, or a busy walkway or plaza, the learner can go out of the classroom and engage the general public in practicing basic interviewing skills and piloting questions from a data collection instrument. For example, the learner can collect data to bring back to the classroom, such as whether people are wearing hats, so they can create a collective distribution diagram during a biostatistics lesson. A specific and somewhat tight time limit should be given with the understanding that this is a part of the training, not a break.

### Presentations from the field

#### Small or large groups

Where instruction is being given to individuals representing a variety of expertise levels, presentations by two or three learners who have more experience or expertise can provide a highly educational perspective. For example, in a training we carried out on strengthening population-based influenza surveillance, influenza officers from countries with a strong surveillance system were asked ahead of time to present on the design, site selection, case selection, and limitations or barriers in their surveillance systems. Other learners, on hearing the presentations, asked specific logistical and operational questions and observed how concepts being taught in a lecture really did apply to the “real world.” This helps build professional networks that learners can turn to for expertise or advice post-training.

## Discussion

Much like the practice of field epidemiology itself, there is an art and a science to producing a case study. We lay out a process for structuring a case study around key teaching points, finding elements to include in a case study plot, and incorporating interactive activities and methods throughout a case study, acknowledging that real-world situations may not always follow a predefined sequence.

First, ensure that the goals and content of the training are compatible with the goals of a case study mentioned earlier in this paper. Case studies lend themselves well to situations in which the learners have some experience working in their designated fields to enhance their participation. Clear didactic materials (whether lecture, job aid, or other format) for learners to refer to can keep the discussion focused appropriately. When a group of learners comes together, the discussion can flounder or become derailed by lack of clarity in the underlying concepts. Thus, it is important that any didactic materials also be carefully crafted. An invited or guest speaker for didactic materials can be beneficial but can also confuse learners if concepts are presented differently than in the case study or other training materials. If guest speakers are delivering didactic content, we have found that it is easiest to provide them slides covering the critical points. This ensures the speaker meets the required objectives, and provides a benefit to the course as the speaker can give subject matter feedback to curriculum developers. When teaching complex or involved ideas, we have found that having an additional checklist or conceptual aid handy helps keep the discussion on track (and the training on time).

Second, consider the amount of time for the training, characteristics of the learners and training content, and select a format. For long trainings, change the case study format to prevent learner fatigue. However, do think about contextual factors when selecting a format. In one multi-day training we carried out, learners were to complete a “go-out” assignment. However, this assignment was near the end of the week (learners were tired), and we chose an hour just before lunch to complete the assignment so that students would be able to interact with a larger number of patrons at the cafeteria at the training site. However, most of our learners were distracted by a coffee and a snack. While they eventually completed the assignment, we lost almost an hour of training time. Other considerations for choosing a format include number of participants, level of learner expertise, cultural sensibilities in interacting with one another, and availability of teaching and support staff.

Third, identify key steps and teaching points for inclusion as questions or processes during the case study. If the training content is not easily related to a set of steps or a process, it should be tailored to the identified learning objectives and should ensure that the “action” verbs from learning objectives are carried out. Write questions around these points that coincide with the plot or unfolding of the scenario. Professional experience or published literature can provide a basis for scenario development.

Fourth, ensure that questions are clear and action-oriented. Always provide an answer key or main teaching points that should be derived from each question in an instructor copy. Estimate that it will take about 10 min for a group to read, discuss, and record answers to each question.

In settings where breakout groups are utilized, it is recommended there be a facilitator embedded with each group to help moderate, ensure all have a chance to participate, that appropriate effort is being exerted, and that learners generally arrive at the intended answers.

## Conclusion

Participatory case studies are a beneficial way of delivering training for professionals who need to learn, reinforce, and apply specific skills to carry out their job duties. In the field of Public Health, where the responsibilities and hours can be significant, the challenge of recruiting and maintaining dedicated workers must be met in order to protect and promote healthy communities. Training public health professionals to learn new skills and encouraging them to share their experience with others through a network-building group case study is an opportunity that is valuable for our public health work force as well as for our communities.

## Author contributions

AN wrote the manuscript with significant contribution from LB. PM conceptualized the manuscript. All authors provided content expertise, contributed to manuscript revision, read, and approved the submitted version.

### Conflict of interest statement

The authors declare that the research was conducted in the absence of any commercial or financial relationships that could be construed as a potential conflict of interest.

## Footnotes

^1^Fairfax County Virginia: Public Health Nursing at the Fairfax County Health Department. http://www.fairfaxcounty.gov/hd/careers-in-public-health/public-nursing-jobs.htm (2016) (Accessed July 11, 2016).

^2^Summit County Public Health: Careers in Public Health. Careers in Public Health. http://scphoh.org/pages/careers.html (2015) (Accessed July 11, 2016).

^3^IRINNews.org Human Rights: Vaccination teams defeat “Ebola effect” in Guinea (2015). http://www.irinnews.org/news/2015/04/29 (Accessed August 13, 2018).
